# Default in plasma and intestinal IgA responses during acute infection by simian immunodeficiency virus

**DOI:** 10.1186/1742-4690-9-43

**Published:** 2012-05-25

**Authors:** Nada Chaoul, Chantal Burelout, Sandrine Peruchon, Beatrice Nguyen van Buu, Pascale Laurent, Alexis Proust, Martine Raphael, Olivier Garraud, Roger Le Grand, Sophie Prevot, Yolande Richard

**Affiliations:** 1 Commissariat à l’Energie Atomique (CEA), CEA, Institut des Maladies Emergentes et Thérapies Innovantes Service d’Immuno-Virologie, CEA, Fontenay-aux Roses, F-92260, France; 2 Université Paris-Sud, Orsay, F-91060, France; 3 EA3064 Université de Lyon, Faculté de Médecine et Etablissement Français du Sang (EFS) Auvergne-Loire, Saint Etienne, F-42023, France; 4 Service d’Anatomie et Cytologie pathologiques, AP-HP, Hôpital A. Béclère, Clamart, F-92140, France; 5 Université Paris-Sud, Faculté de Médecine, Le Kremlin-Bicêtre, F-94276, France; 6 Service d’Hématologie et Immunologie Biologiques, cytogénétique, CHU-Bicêtre, Assistance Publique-Hôpitaux de Paris (AP-HP), Le Kremlin-Bicêtre, F-94 724, France; 7 INSERM U802, Université Paris-Sud, Le Kremlin-Bicêtre, F-94 724, France; 8 Inserm U1016, Institut Cochin, Paris, France; 9 CNRS UMR8104, Paris, France. Institut Cochin. Département d’Immunologie. 27, Rue du Faubourg St Jacques, Paris, F-75014, France

**Keywords:** Mucosal B-cell response, IgA, HIV/SIV, Germinal centers, BAFF, Terminal ileum

## Abstract

**Background:**

Conflicting results regarding changes in mucosal IgA production or in the proportions of IgA plasma cells in the small and large intestines during HIV-infection have been previously reported. Except in individuals repeatedly exposed to HIV-1 but yet remaining uninfected, HIV-specific IgAs are frequently absent in mucosal secretions from HIV-infected patients. However, little is known about the organization and functionality of mucosal B-cell follicles in acute HIV/SIV infection during which a T-dependent IgA response should have been initiated. In the present study, we evaluated changes in B-cell and T-cell subsets as well as the extent of apoptosis and class-specific plasma cells in Peyer’s Patches, isolated lymphoid follicles, and lamina propria. Plasma levels of IgA, BAFF and APRIL were also determined.

**Results:**

Plasma IgA level was reduced by 46% by 28 days post infection (dpi), and no IgA plasma cells were found within germinal centers of Peyer’s Patches and isolated lymphoid follicles. This lack of a T-dependent IgA response occurs although germinal centers remained functional with no sign of follicular damage, while a prolonged survival of follicular CD4+ T-cells and normal generation of IgG plasma cells is observed. Whereas the average plasma BAFF level was increased by 4.5-fold and total plasma cells were 1.7 to 1.9-fold more numerous in the lamina propria, the relative proportion of IgA plasma cells in this effector site was reduced by 19% (duodemun) to 35% (ileum) at 28 dpi.

**Conclusion:**

Our data provide evidence that SIV is unable to initiate a T-dependent IgA response during the acute phase of infection and favors the production of IgG (ileum) or IgM (duodenum) plasma cells at the expense of IgA plasma cells. Therefore, an early and generalized default in IgA production takes place during the acute of phase of HIV/SIV infection, which might impair not only the virus-specific antibody response but also IgA responses to other pathogens and vaccines as well. Understanding the mechanisms that impair IgA production during acute HIV/SIV infection is crucial to improve virus-specific response in mucosa and control microbial translocation.

## Background

The gastrointestinal tract is a privileged site for both HIV-1/SIV replication and extensive CD4^+^ T-cell depletion at all stages of the pathogenic infection [1,2]. One of the physiological roles of the gastrointestinal tract in immunological defense is to produce large amounts of IgA that contribute to the protection of the intestinal mucosa from pathogens [3]. IgA are produced from plasmablasts (plasma cell precursors) generated in germinal centers (GC) of Peyer’s patches (PP) and mesenteric lymph nodes that constitute major inductive sites of T-dependent IgA antibodies. Both T-cell help and local production of cytokines participate in T-dependent IgA production [4-7]. IgA class switching also occurs in isolated lymphoid follicles (ILF), which have a cellular composition similar to PP-associated follicles and constitute dynamic lymphoid structures that develop in response to chronic infection or inflammation [8-10]. In addition to the canonical TGFβ1 IgA switch factor, IL10, IL21, B-cell Activating Factor of the TNF Family (BAFF) and A Proliferation-Inducing Ligand (APRIL) are also key factors involved in both T-dependent and T-independent immunoglobulin class switching [9,11]. These latter cytokines most likely account for IgA production in children with defective CD40L [12,13]. In contrast to humans, only one IgA isotype is found in macaques, with a structure resembling that of human IgA2 [14,15].

Although conflicting results exist concerning the presence of HIV-specific IgA in genital secretions of women repeatedly exposed to HIV-1 but yet remaining uninfected [16-19], HIV-specific IgA are generally absent or present at very low levels in plasma and mucosal secretions of chronically HIV-infected patients [20-24]. In contrast to other encountered mucosal microbial infections, HIV-1 infection preferentially leads to potent IgG responses in any body fluids. Decreased levels of IgA in intestinal fluids associates with reduced proportions of IgA plasma cells within the lamina propria (LP) of the duodenum and colon of chronically HIV-infected patients [25]. In AIDS patients, depletion of IgA plasma cells in the small intestine also correlates with decreased secretion of IgA in saliva [22]. In supernatants of short-term cultured duodenal biopsies from SIV-infected macaques, Schafer at al. described a decrease in total IgA and a lack of SIV-specific IgA for up to 6 months post-infection [26]. However, measuring HIV/SIV-specific and total antibodies in intestinal and vaginal secretions by ELISA constitutes a technical challenge [27], likely contributing to conflicting results. Moreover, IgA secretion is highly dependent on the integrity of epithelial cells, which is frequently impaired during acute and chronic HIV/SIV infections [28-30]. Overall, the lack of IgA in intestinal fluids of chronically HIV-infected patients or SIV-infected macaques can be explained by a default in shedding, impaired homing of IgA plasmablasts to LP or their impaired terminal differentiation into plasma cells. In addition, recent data of Xu et al. suggested that an impaired isotype switching towards IgA occurs in inductive sites [31], preventing the generation of IgA plasmablasts.

Here, we have examined changes in the proportions of IgA vs. IgG and IgM plasma cells within the inductive sites (germinal centers of B-cell follicles) and effector site (LP) of the small intestine (duodenum and terminal ileum) of acutely SIV-infected macaques to determine at which step the generation of IgA plasma cells was impaired. Our data reveal a progressive decrease in plasma IgA after SIV infection, associated with a lack of IgA plasma cells within GC of PP and isolated lymphoid follicles while functional GC can still give rise to IgG plasma cells. Whereas the densities of total plasma cells increased in the LP, the relative proportion of IgA plasma cells decreased. Altogether, these findings suggest that SIV is unable to initiate a T-dependent IgA response during the acute phase of SIV infection despite prolonged survival of follicular CD4^+^ T-cells.

## Results

### Increased B-cell homing to the intestinal mucosa during the acute phase of infection

**Figure 1:**
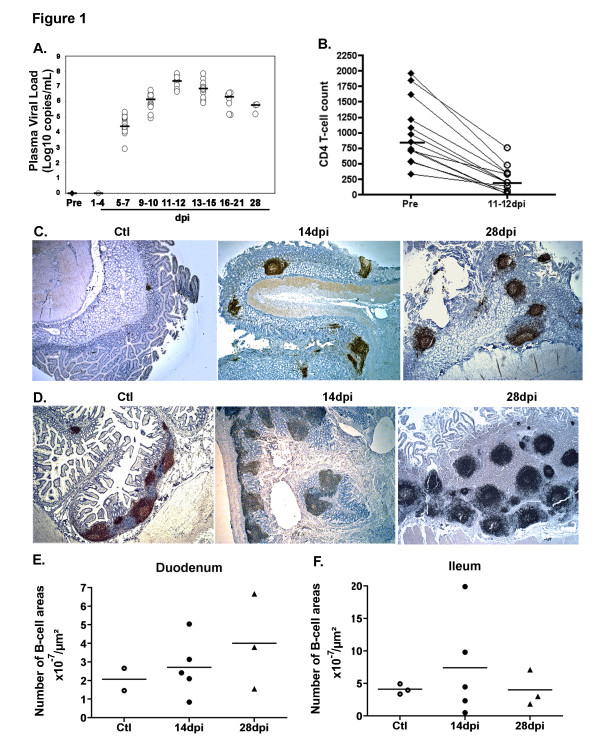
**Increased number of B-cell areas in the intestinal mucosa in acutely SIV-infected macaques.** (**A**) For each of the 13 SIV-infected macaques, viral load was measured in plasma before infection (Pre; filled circles) and every 3 dpi (open circles). Results are expressed as Log10 copies/mL and bars represent median values. (**B**) CD4^+^ T-cells were quantified in whole blood collected from each macaque before infection and every 3 dpi. For each animal, CD4^+^ T-cell counts before infection (filled circles; n=13) and at 11-12 dpi (open circles, n=13) are shown. Results are expressed as cells/μL and bars represent median values. *** *p*=0.0004 (Wilcoxon test) (**C-D**) Staining by CD20 mAb of sections from duodenum (**C**) and terminal ileum (**D**) of one representative non-infected macaque (Ctl, n=3) and macaques infected for 14 (14 dpi, n=5) or 28 days (28 dpi, n=3). Magnification x25 for all panels (**E-F**) The number of CD20^+^ B-cell areas per section was divided by the surface of the total mucosa. Results are expressed as B-cell areas x 10^-7^/μm^2^. Each symbol corresponds to one animal. Bars represent median values for each group.

Both changes in levels of plasma viral load and CD4^+^ T-cell count followed similar kinetics and range in all macaques. By 11–12 dpi, plasma viral load peaked with a median value of 7.36 log_10_ SIV RNA copies/ml before decreasing to 5.78 log_10_ SIV RNA copies/ml on 28 dpi (Figure 1A). As compared to its value before infection, the median blood CD4^+^ T-cell count reached its lowest value on 11–12 dpi (76.9% decrease, *p* = 0.0004), coincident with the peak in viral load (Figure 1B). In agreement with our previous observations [32], SIV-infection rapidly induced B-cell accumulation in the intestinal mucosa as compared to controls (Figure 1C1D). However, the intensity of B-cell infiltration was variable among SIV-infected animals and even at different sites of the intestinal mucosa within one animal (Figure 1E and 1F). The median number of B-cell areas per μm^2^ of total duodenal mucosa increased by 1.2-fold and 1.8-fold on 14 and 28 dpi, respectively (Figure 1E) whereas it increased by 1.1-fold and 1-fold in the terminal ileum, respectively (Figure 1F). Despite high numbers of isolated B-cell follicles in terminal ileum of SIV-infected animals, the concomitant increase in mucosal thickness lowers the frequency value. (Figure 1D, right panel). The development of GC within B-cell follicles constitutes an independent marker of B-cell activation. Within the intestinal mucosa of SIV-infected animals, B-cell follicles rapidly developed GC, with 39% of duodenal follicles containing GC as compared to 3% in controls. Whereas an average of 21% ileal B-cell areas contained GC in controls, 34% and 44% of them contained GC at 14 and 28 dpi, respectively.

These data evidence a consistent B-cell activation in intestinal mucosa during acute SIV infection, with an early development of GC in intestinal B-cell follicles, which is indicative of the initiation of TD B-cell response. However, SIV-specific antibodies are only detectable in plasma from 21 dpi on in these animals [29].

### Preferential increase in IgG plasma cells within GC during acute SIV infection

Because CD20 expression is rapidly lost during terminal B-cell differentiation, human plasma cells and their immediate precursors are generally detected using CD19, CD38 and CD138 mAb. While CD19 expression progressively decreases, CD38 expression begins to be upregulated on human plasmablasts and CD138 expression characterizes late stages of plasma cell maturation [33]. The detection of macaque B-cells engaged into terminal differentiation is limited by the lack of suitable reagents: human CD19 and human CD138 mAb possess limited and no cross-reactivity, respectively, while human CD38 mAb are unable to discriminate plasma cells from other simian B-cells subsets (*data not shown).* We thus evaluated changes in plasma cells by immunohistochemistry using alternative markers: IRF4/MUM-1 and cytoplasmic Ig. High IRF4/MUM-1 protein expression is a hallmark of normal plasma cell differentiation and a major marker of myeloma cells [34].

**Figure 2:**
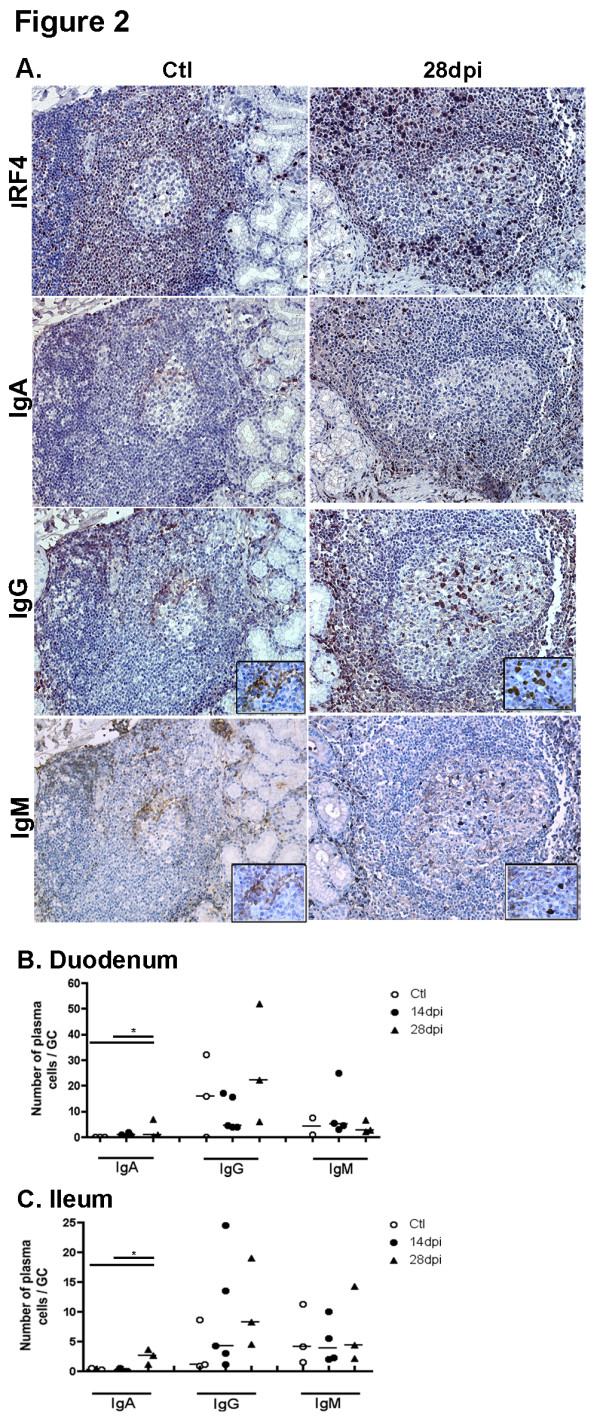
**Acute SIV infection induces a dominant IgG/M over IgA response in GC. (A)** Duodenal sections from controls (Ctl left panels) and macaques infected for 28 days (28 dpi, right panels) were stained with IRF4 mAb, IgA, IgG and IgM polyclonal Ab. Staining of one representative section for each group of macaques is shown. Magnification x200 and x1000 for enlarged images. (**B-C**) Each symbol represents the mean number of IgA, IgG and IgM plasma cells per GC in duodenum (**B**) and terminal ileum (**C**) in one macaque. Bars represent median values for each group.

We found rare IgA plasma cells in GC of the duodenal mucosa of both control and SIV-infected macaques. In contrast, total (stained by IRF4 mAb) and IgG plasma cells per GC were 1.1- and 1.4-fold more numerous in SIV-infected macaques on 28 dpi than in controls (Figure 2A). In terminal ileum, the median number of total plasma cells increased by 1.9- and 3.1-fold on 14 and 28 dpi, respectively. This increase was essentially due to increased numbers of IgG plasma cells per GC, with a 3.8- and 7.2-fold increase on 14 and 28 dpi, respectively (Figure 2C). Therefore, SIV-infection preferentially promotes an IgG response within GC of the small intestine as in spleen and mesenteric lymph nodes [32].

### SIV infection modifies the ratio of IgA versus IgM or IgG plasma cells within the LP

**Figure 3:**
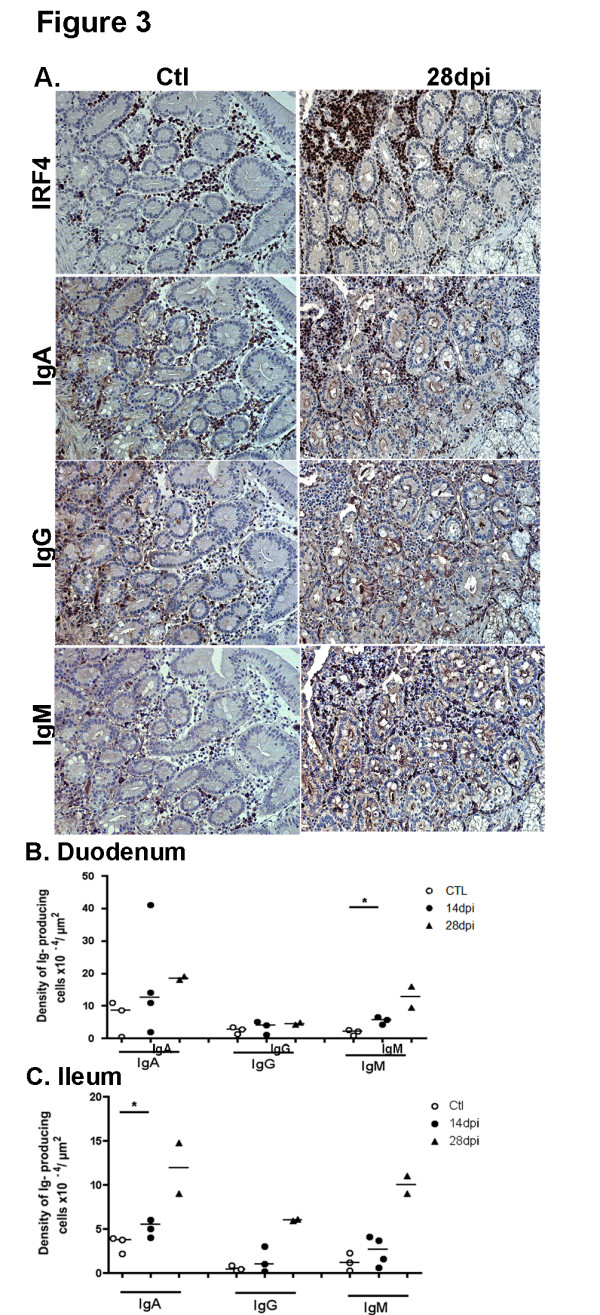
**Acute SIV infection induces an increase in total plasma cells in the intestinal LP with a dominant IgG/M over IgA response. (A)** Duodenal sections from controls (Ctl left panels) and macaques infected for 28 days (28 dpi, right panels) were stained with IRF4 mAb, IgA, IgG and IgM polyclonal Ab. Staining of one representative section for each group of macaques is shown. Magnification x200 and x1000 for enlarged images. (**B, C**) Each symbol corresponds to the mean number of IgA, IgG and IgM plasma cells in LP of duodenum (**B**) and terminal ileum (**C**) from one macaque. Bars represent median values for each group. * *p* values <0.05 (Mann–Whitney, one tailed).

Comparison between median values of each group showed that densities in total plasma cells in the duodenal LP were 1.6- and 2.6-fold higher on 14 and 28 dpi, respectively, than in controls (Figure 3A). However, a variable increase in the density of each class-specific plasma cells occurred on 14 and 28 dpi: 2.6- and 5.9-fold increase for IgM plasma cells, 1.4- and 2.1-fold increase for IgA, and 1.4- and 1.6-fold increase for IgG, respectively (Figure 3B). Therefore, higher proportions of IgM plasma cells were observed on 28 dpi (36% vs. 16% in controls, 2.2-fold) at the expense of IgG (13% vs. 21% in controls) and IgA (52% vs. 63% in controls) plasma cells (Additional file 1: Figure S1A).

In the terminal ileum, the density of total plasma cells also increased in the LP by 1.7- and 4.9-fold on 14 and 28 dpi as compared to controls (Figure 3C). As also compared to controls, the density of IgA plasma cells increased by 1.4- and 3.1-fold on 14 and 28 dpi, that of IgG by 2.5- and 15-fold and that of IgM by 2.2- and 8.3-fold, respectively. These changes resulted in higher proportions of IgG plasma cells at 28 dpi (21.5% vs. 7%) at the expense of IgA plasma cells (43% vs. 66%) (Additional file 1: Figure S1B). These data show that SIV-infection favors the generation of IgM (duodenum) or IgG (ileum) rather than IgA plasma cells in the LP of the small intestine.

Taken altogether these data suggest a biased isotype class switching or an impaired survival of IgA plasma cells in the intestinal mucosa of acutely SIV-infected macaques.

### Differential survival of CD4^+^ CD45RO^+^ T-cells in GC, follicular T-cell zones and LP

**Figure 4:**
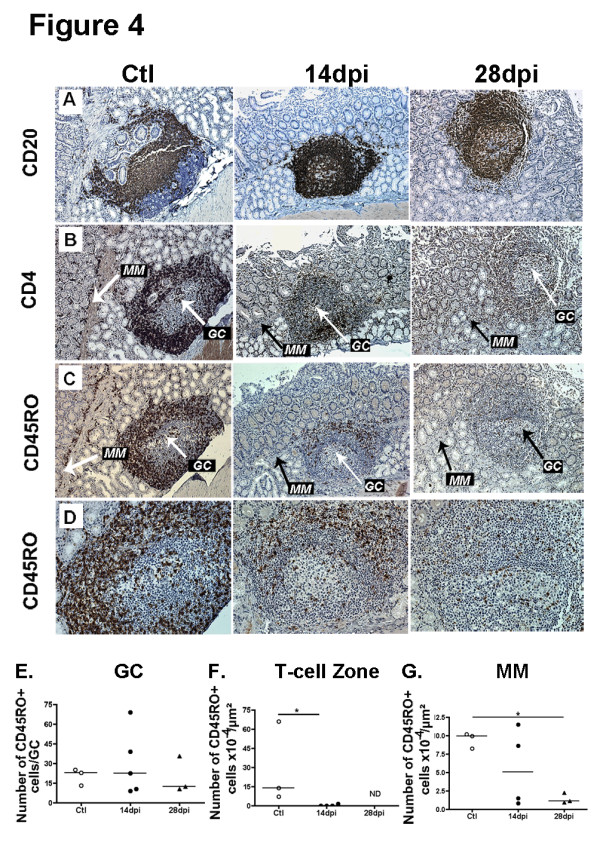
**Prolonged survival of CD4 + CD45RO + T-cells in GC as compared to follicular T-cell zones and lamina propria.** (**A-D**) Duodenal sections from controls (Ctl) and macaques infected at 14dpi and 28dpi were stained with CD20 mAb (**A**), CD4 mAb (**B**), CD45RO mAb (**C-D**). In **A** to **D**, data from one representative macaque per group are shown. Original magnification x100 for panels A to C, x200 for panel D. (**E-G**) Each symbol corresponds to the mean density of CD45RO^+^ cells per μm2 of duodenal *muscularis mucosae (MM,****G****),* of follicular T-cell zone (**F**) and to the mean number of CD45R0^+^ cells per GC (**E**) per macaque*.* Bars represent median values for each group. * *p* values <0.05 (Mann–Whitney).

We next examined T-cell changes in the LP and inductive sites (PP and ILF) of the small intestine of SIV-infected macaques. In the duodenum of control animals, we observed numerous CD4^+^ (Figure 4B) and CD45RO^+^ T-cells (recognized by the OPD4 clone) (Figure 4C,D) distributed throughout the LP as well as in follicular T-cell zones. As compared to control macaques, CD45RO^+^ T-cell density was 80% lower in the follicular T-cell zones at 14 dpi (Figure 4F). The well-defined line of CD4^+^ and CD45RO^+^ T-cells, visible at the interface between mucosa and *muscularis mucosae* in controls (Figure 4B, C, left panels, arrow) was no more detectable in SIV-infected macaques, except near and above B-cell follicles (Figure 4C, arrow); indeed CD45RO^+^ T-cell density was decreased by 78% and 88% in *muscularis mucosae* at 14 and 28 dpi, respectively (Figure 4G). In contrast, the frequency of CD45RO^+^ T-cells within the GC of duodenal B-cell follicles did not decrease before 28 dpi (44% decrease) (Figure 4E). As shown by CD23 and Ki67 staining, the polarization of GC was preserved until 28 dpi (Additional file 2: Figure S2).

In the terminal ileum, the median CD45RO^+^ T-cells density decreased by 59% and 73% respectively at 14 and 28 dpi in follicular T-cell zones, and by 84% in *muscularis mucosae* at 14 dpi*,* as compared to controls (*data not shown*). Concomitantly, a 1.8-fold increase in CD45RO^+^ T-cells was observed within the GC of ileal B-cell follicles on 14 dpi. Thus, in contrast to follicular T-cell zones and *muscularis mucosae* where the depletion in CD4^+^ T-cells rapidly progressed from 14 dpi on, CD4^+^ CD45RO^+^ T-cells accumulated within GC of mucosal B-cell follicles at 14 dpi.

### Distinct kinetics of apoptosis in GC, follicular T-cell zones and LP

**Figure 5:**
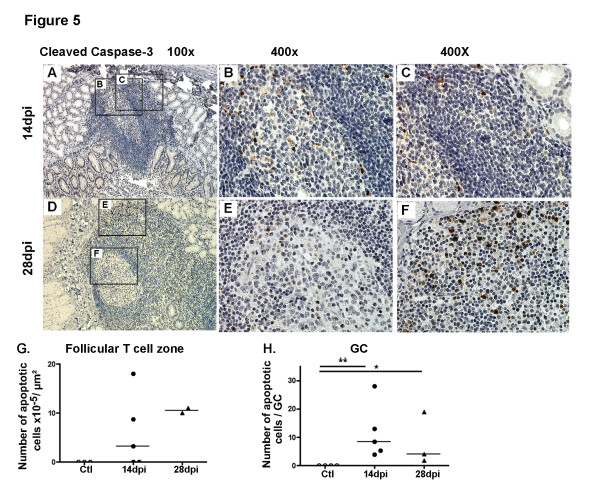
**Apoptosis progresses more rapidly in LP than in GC. (A-F)** Duodenal sections from one representative macaque at 14 dpi and 28 dpi were stained with anti-cleaved caspase-3 Ab (**A-F**). Inserts from left panels are shown in middle (B and E; showing GC) and right panels (C and F; showing follicular T-cell zones). Original magnification x100 in left panels and x400 in middle and right panels. (**G-H**) Each symbol corresponds to the mean density of cleaved caspase3+ cells per μm^2^ of follicular T-cell Zone (**G***) and* to the mean number of cleaved caspase-3^+^ cells per GC (**H**) per macaque*.* Bars represent median values for each group. *p value < 0.05 (Mann–Whitney).

Because increased apoptosis might contribute to the paucity of IgA plasma cells, we examined the expression of the cleaved caspase-3, a key mediator of apoptosis shared by membrane- and mitochondrial-mediated pathways. In the duodenal mucosa, no cleaved caspase-3^+^ cells were found within GC or follicular T-cell zones of control macaques (data not shown). In the follicular T-cell zone, the median density increased by 6.5-fold between 14 and 28 dpi (Figure 5A-F, 5 G) whereas the median number of cleaved caspase-3+ cells is 10.5 and 3.2 cells/GC at 14 and 28 dpi, respectively (Figure 5H). In the terminal ileum, the median density of apoptotic cells increased by 11-fold in follicular T-cell zones and by only 1.7-fold in GC at 14 dpi, as compared to controls (data not shown). These results indicate that apoptosis progresses with different kinetics in the inductive and effector mucosal compartments of SIV-infected animals.

### Decrease in plasma IgA despite increased BAFF production

**Figure 6:**
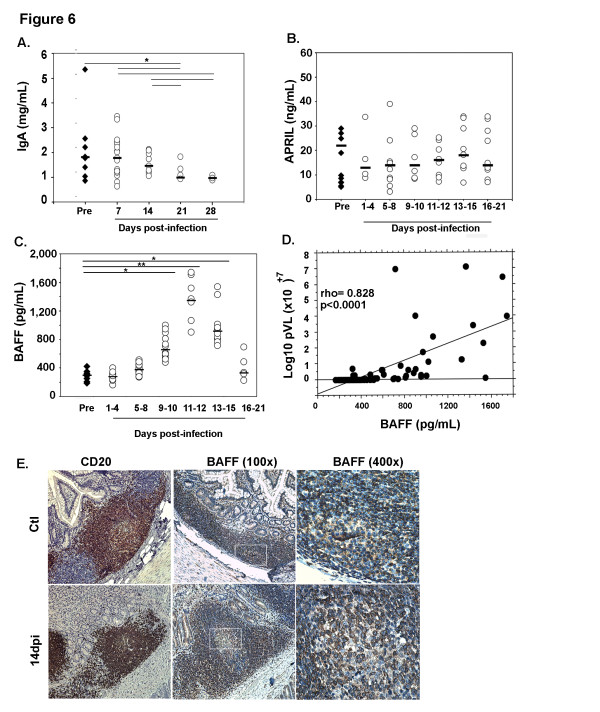
**Decreased plasma IgA levels despite a strong increase in BAFF levels.** (**A**) Plasma IgA levels were measured in SIV-infected macaques before infection (Pre; filled diamonds) and every 7 dpi (open circles). Results are expressed in mg/mL and bars represent median levels. Significant differences between groups are shown (*p < 0.05). (**B-C**) APRIL and BAFF levels were measured in plasma from SIV-infected macaques, before (Pre: filled diamonds) and every 3 dpi (open circles). Results are expressed in pg/mL (BAFF) and ng/mL (APRIL), and bars represent median levels. Significant differences between groups are shown (*p < 0.05, **p < 0.01). (**D**) Correlation between BAFF levels and plasma viral load (Log10 pVL) is shown. (**E**) Ileum sections from control macaques (upper panel) and macaques infected for 14 days (lower panel) were stained with anti-CD20 (B-cells, left panels) or anti-BAFF (Buffy2, middle and right panels) mAb. Original magnification: x200 for CD20, x100 and x400 for Buffy2.

In contrast to increased plasma IgG and IgM levels observed in these macaques prior to 14 dpi [32], plasma IgA levels progressively decreased from 18% on 14 dpi to 46% on 28 dpi as compared to baseline values before infection (Figure 6A). Considering that BAFF and APRIL are important IgA inducing factors [13,35-37], we measured both of these cytokines in plasma. The median concentration of plasma APRIL, which was 22 ng/ml before infection, varied between 13 and 18 ng/ml after SIV infection (Figure 6B). In contrast, we observed a sharp increase in plasma BAFF that peaked at 11–12 dpi (1,347 pg/ml versus 298 pg/ml before infection, 4.5-fold increase), and remained elevated at 13–15 dpi (918 pg/ml, 3.1-fold increase) and decreased toward baseline values thereafter (Figure 6C). Throughout the acute phase of infection, BAFF levels correlated with plasma viral load (*p* < 0.0001) (Figure 6D) and inversely with circulating CD4^+^ T-cell counts *(rho = −0.596, p* < 0.0001) but not with plasma IgA (*data not shown*).

In an attempt to correlate increased plasma BAFF levels with its local production in lymphoid tissues, we compared BAFF expression in the intestinal mucosa of acutely SIV-infected and control macaques using Buffy2 mAb and immunohistochemical approach. Both isolated cells and stromal cell network associated with B-cell follicles, and GC were strongly stained by Buffy2 mAb (Figure 6E). The stromal network in T-cell areas immediately adjacent to B-cell follicles was also BAFF^+^. Our present results thus reveal increased BAFF production in intestinal mucosa even if additional experiments in SIV-infected macaques are needed to identify BAFF-producing cells in this microenvironment during SIV infection as well as to quantify the magnitude of BAFF overproduction in specific tissue areas. Thus, plasma IgA progressively decreases during acute SIV infection despite increased systemic and mucosal production of BAFF.

## Discussion

Dysfunctions in circulating B-cells have been extensively described in chronically, and to a lesser extent, in primary HIV-infected patients [38-40]; but only very few studies have been devoted to mucosal B-cells during primary HIV infection [41]. This situation contrasts with the pivotal role of the intestinal mucosa as a major site of IgA response in healthy individuals as well as a site of intense and early virus replication, heavy damages to CD4^+^ T-cell subsets and chronic inflammation in HIV-infected individuals. Consistent with early inflammation described in pathogenic SIV/HIV infection, we observed overproduction of BAFF and infiltration of the intestinal mucosa by isolated lymphoid follicles, which progressively organized into secondary follicles containing well-developed GC (32 and this work). This increase in B-cell areas is consistent with increased proportions of B-cells within the ileal LP of primary HIV-infected patients reported by Levesque et al. [41]. In addition to PP, isolated lymphoid follicles constitute important sites for the IgA response against bacteria and are also associated with chronic inflammation in several disorders [42,43]. As previously observed in mesenteric lymph nodes of the same animals [32], a dominant IgG over IgA response was observed in GC of both PP and isolated lymphoid follicles. The sustained generation of IgG plasmablasts in these GC that display a preserved polarization ([32] and Additional file 2: Figure S2) is in favor of functional GC, which nevertheless have lost the capacity to support T-dependent IgA response. In SIV-infected macaques, normal expression of Bcl6 and Ki67 by GC B-cells (Additional file 2: Figure S2) suggests that decreased production of IL21 by follicular helper T-cells is likely not responsible for the lack of IgA plasma cells. The expression of these factors is under the control of IL21-IL21R interactions [5,44]. Because most systemic IgA are of T-dependent origin, the marked decrease in plasma IgA levels is consistent with the absence of IgA plasmablasts within GC. However, the precise mechanism underlying their absence within functional GC remains to be further investigated.

As compared to their accelerated death in LP and follicular T-cell zones, CD4^+^ CD45RO^+^ T-cells were present within GC until 28 dpi, suggesting a prolonged survival within this special environment. This observation could be related to the low CCR5 expression on follicular helper T-cells, which might protect them from SIV infection [45]. A less dramatic loss of CD4^+^ T-cells in PP than in LP has been previously reported in SIV-infected monkeys [2,46]. Moreover, Zhang et al. have shown that early GC disruption (20 dpi) occurs preferentially after SIV infection of Indian rhesus monkeys, a model of rapid disease progression [47]. Aside from the results of Levesque et al. showing GC fragmentation in primary HIV-infected patients [39], involution of GC is more frequent during chronic and advanced phases of the disease when CXCR4 variants are present [31,48]. However, treatment of HIV-infected patients with CCR5 antagonists has shown that small populations of X4 variants are more frequently present during primary HIV infection than previously suspected [49]. When present, these variants might contribute to the infection of follicular helper T-cells expressing CXCR4 and, hence, to their apoptosis [45].

Because of the presence of follicular helper T-cells and appropriate GC organization, other mechanisms could account for the lack of T-dependent IgA response within GC. Among other possibilities, a decreased production of active TGFβ1 or retinoic acids associated with impaired activation of intestinal dendritic cells might reduce the IgA response [50,51]. Interestingly, differential kinetics of TGFβ1 production and responsiveness to TGFβ1 distinguish pathogenic from non-pathogenic SIV infections [52,53]. The frequency of TGFβ-positive cells also increases in the peripheral or mesenteric lymph nodes of SIVmac-infected rhesus macaques but not in those of African green monkeys or sooty mangabeys [54,55]. Even in acutely SIV_mac_-infected macaques where TGFβ^+^ cells accumulate in paracortical T-cell zones of lymph nodes with a peak at 11-16dpi, these cells were absent from GC of B-cell follicles. Whether the lack of TGFβ^+^ cells reflects impaired T-cell production in SIV-infected animals or normal low levels of TGF within GC remains to be clarified. In addition, the absence of newly generated IgA plasmablasts at the benefit of IgG and IgM ones as well as the rapid loss of CD4^+^ T-cells within the LP, likely participates to decrease the proportions of IgA plasma cells in this effector site.

## Conclusion

Our data point to a generalized default in IgA response, already detectable during the acute phase of infection. The understanding of mechanisms causing this impaired IgA response during HIV/SIV infection is crucial in order to improve the mucosal virus-specific response and control microbial translocation. Considering the importance of mucosal defenses in determining the host-pathogen balanced relationship [56-58], it remains to be established whether the early default in the T-dependent IgA response similarly occurs after infection by mucosal routes which represent the more frequent routes of HIV infection.

## Methods

### Animals and ethics statement

Mauritian adult male Cynomolgus macaques (*Macaca fascicularis*), weighing 4 to 6 kg, were housed each in single cages within level 3 biosafety facilities. Animals were housed and cared for in accordance with the European Guidelines for Animal Care (“*Journal officiel des Communautés Européennes*” L358, 18 décembre 1986). A regional Animal Care and Use Committee: “Comité Régional d’Ethique sur l’expérimentation animale Ile-de-France Sud”, reviewed and approved all protocols, with the goal of improving animal welfare and limiting unnecessary suffering. The animals were sedated with Ketamine chlorydrate (Rhone-Merieux, Lyon, France) before virus injection, blood sample collection or euthanasia. The animals were inoculated intravenously with 50 AID50 SIVmac251 and were euthanized at 14 (five animals), 21 (five animals) and 28 (three animals) days post-infection (dpi). Blood samples were collected before infection and every 3 dpi thereafter until euthanasia. Plasma samples were kept at −80°C until use. Changes in plasma viral load, CD4^+^ T-cell counts, circulating and tissue-specific B-cell subsets of these SIV-infected macaques have been previously reported [32]. After euthanasia, duodenal and terminal ileum tissue samples (corresponding to a five cm section before the caecum) were formalin-fixed and paraffin-embedded. Control tissues were collected from three non-infected animals.

### Quantification of immunoglobulin A and cytokines

Plasma BAFF and APRIL levels were respectively quantified using the Quantikine® ELISA kit for human BAFF/BLyS (R&D systems, Abingdon, UK) and APRIL human ELISA from Bender Medsystems (Tebu-Bio, Le Perray en Yvelines, France) according to the manufacturer’s instructions. Plasma IgA concentrations were determined using a monkey IgA ELISA kit (Alpha diagnostic Intl Inc., San Antonio, Tx). Each sample was run in duplicate and results expressed as mean concentration (pg/mL for BAFF, ng/mL for APRIL and mg/ml for IgA) ± SD for each animal and at each time point.

### Immunohistochemistry (IHC) and image analysis

All paraffin-embedded tissues were cut into (3 μm) sections, perpendicularly to the intestinal wall so that each intestinal compartment could be examined. After antigen retrieval in sodium citrate (pH6) (CD20, Ki67, Buffy2), EDTA (pH 9) (CD3) or EDTA (pH 8) (other Ab), sections were then labeled with optimized concentrations of monoclonal or polyclonal antibodies against: CD20 (L26), IRF4 (MUM-1P), IgA (rabbit F(ab’)_2_, IgG (rabbit F(ab’)_2_, IgM (rabbit F(ab’)_2_, CD45R0 (OPD4), and CD68 (clone KP1) (all from Dako, Glosturp, Danemark), CD4 (1 F6) and CD23 (1B12) (from Novocastra, Newcastle, UK), Ki67 (MIB5, Beckman Coulter, Fullerton), CD3 (SP34-2, Becton Dickinson, Franklin Lakes, NJ) and cleaved caspase-3 (rabbit serum, Cell Signaling Technology Inc., Danvers, MA). Polyclonal Ig and mouse isotype controls were from Dako or R&D systems. Antibody binding was visualized with the Novolink anti-rabbit/mouse secondary Ab Polymer kit (Novocastra Laboratories, Newcastle upon Tyne, UK) according to the manufacturer’s instructions. Binding of Buffy2 mAb (Enzo life Sciences, Villeurbanne, France) was visualized by the *EnVision™ +* Dual Link Kit from Dako. Nuclei were counter-stained with Hematoxylin QS (Vector Laboratories, Burlingame, CA). Digital images of tissue sections were captured without manipulation using a Zeiss Microscope (Axiophot 2) coupled to a Microfire camera (Optronics, CA) and using the MorphoLite software (Explora Nova, La Rochelle, France).

Image analysis was performed with the Mercator 4.42 software (Explora Nova) on tissue sections from three non-infected animals as controls and from SIV-infected animals euthanized at either 14 or 28 dpi. Epithelium was outlined by hand and excluded in each field. Area from *muscularis mucosae* to tips of villi was referred to as “total mucosa” whereas CD3^+^ T-cell areas surrounding B-cell areas, including isolated lymphoid follicles and B-cell follicles in PP, were referred to as “follicular T-cell zones” throughout the manuscript. The LP was defined as total mucosa minus follicular T-cell zones and B-cell areas. The number of B-cell areas was determined by counting CD20^+^ areas in total mucosa at 100X magnification, corresponding to surface areas of 9,467 to 308,392 (duodenum) and 8,000 to 12,409 (ileum) μm^2^. Data are expressed as the average number of B-cell areas per μm^2^ of total mucosa. Immunoreactive cells were counted in all GC present in every section at 100X magnification. By using the Novolink anti-rabbit/mouse secondary Ab Polymer kit, we experienced limited difficulties in discriminating Ig-producing cells from the reticular background possibly observed in GC or sub-epithelial areas. Accordingly, only cells with a clear cytoplasmic staining were taken into account for quantification. Data are expressed as mean (±SD) number of positive cells per GC. Immunoreactive cells were counted in follicular T-cell zones, *muscularis mucosae (MM)* or LP by analyzing as many fields as possible at 100X magnification. Surface areas of 7,900 to 112,172 (duodenum) and 6,167 to 161,140 (ileum) μm^2^ for follicular T-cell zones; 1,182 to 71,742 (duodenum) and 29,000 to 51,500 (ileum) μm^2^ for *muscularis mucosae*; 147,000 to 900,000 (duodenum) and 235,000 to 750,000 (Ileum) μm^2^ for LP were analyzed. Data are expressed as the mean density of immunoreactive cells (number of positive cells (±SD)/μm^2^).

### Statistical analyses

Non-parametric Wilcoxon‘s test or Mann-Whitney’s test (two-tailed unless otherwise indicated) and correlations (Spearman’s rank test) were assessed using the GraphPad Prism 5 software (La Jolla, CA). *p* values < 0.05 were considered as significant.

## Abbreviations

Ab, antibody (ies); APRIL, a proliferation-Inducing ligand; BAFF, B-cell activating factor of the TNF family; Dpi, days post-infection; GC, germinal center(s); HIV, human Immunodeficiency Virus; Ig, immunoglobulin(s); ILF, isolated lymphoid follicles; LP, lamina propria; PP, Peyer’s Patches; SIV, Simian Immunodeficiency Virus.

## Competing interests

The authors declare that they have no competing interests.

## Authors’ contributions

NC, CB and SP carried out ELISA and flow cytometric surface analyses. NC, BNVB, PL and AP performed immunohistochemical analyses and quantifications. NC, CB, SP and YR analyzed the data and wrote the paper. NC, CB, MR, OG and YR reviewed the paper. YR designed and coordinated the study. RLG, OG and YR edited the manuscript. All authors read and approved the final manuscript.

## Authors’ details

^1^CEA, Institut des Maladies Emergentes et Thérapies Innovantes, Service d’Immuno-Virologie, F-92260-Fontenay-aux Roses, France; ^2^ UMR-E1, Université Paris-Sud, F-91060 Orsay, France; ^3^ EA3064 Université de Lyon, Faculté de Médecine et Etablissement Français du Sang (EFS) Auvergne-Loire, F-42023 Saint Etienne, France; ^4^ Service d’Anatomie et Cytologie pathologiques, AP-HP, Hôpital A. Béclère, F-92140 Clamart, France; ^5^ Université Paris-Sud, Faculté de Médecine F-94276 le Kremlin-Bicêtre, France; ^6^ Service d’Hématologie et Immunologie Biologiques, cytogénétique, CHU-Bicêtre, Assistance Publique-Hôpitaux de Paris (AP-HP), F-94 724 Le Kremlin-Bicêtre, France and ^7^ INSERM U802, Université Paris-Sud, F-94 724 Le Kremlin-Bicêtre, France

## Financial support

This work was supported by grants from the Agence Nationale de Recherche sur le SIDA et les Hépatites Virales (ANRS) (#23-1209 to YR) and the Fondation de la Recherche Médicale (#2006-0306369 to YR). NC and SP were PhD fellows of Europrise Network of Excellence and ANRS, respectively. The Centre National de la Recherche Scientifique (CNRS) supports YR.

## Supplementary Material

Additional file 1 **Figure S1. Acute SIV-Infection changes the density of IgA, IgG and igM plasma cells in the intestinal LP.** The relative proportions of IgA, IgG and IgM plasma cells in the LP of duodenum (A) or ileum (B) were calculated, for each group of macaques, as the ratio between the median numbers of positive cells for one isotype to the median number of total plasma cells X100. Click here for file

Additional file 2 **Figure S2. Preserved polarization of GC in SIV-infected macaques**. (**A-D**) Duodenum sections from controls (Ctl; left panel) and macaques infected for 14 dpi (middle panel) and 28 dpi (right panel) were stained with CD20 (**A**), CD23 (**B**), Ki67 (**C**) and CD68 (**D**) Ab. Stained sections from one representative macaque per group are shown. Original magnification: x100 for all panels. Because T-dependent response is strongly dependent on GC, we analyzed the GC organization after SIV infection. CD23 mAb strongly stains mature FDC network of the light zone, while Ki67 Ab stains proliferating B-cells present in the dark zone and helper T-cells in the light zone. ILF without GC in the duodenal mucosa of controls were stained by CD20 mAb (**A**) but not by Ki67 mAb (**C**). In the absence of typical GC-like structures, CD23 mAb consistently stained the network of stromal cells in these ILF (**B**). After SIV-infection, GC progressively developed in B-cell follicles with numerous Ki67^+^ cells on 14 dpi and a strong staining of a patchy FDC network. On 28 dpi, GCs were clearly hyperplasic but still correctly polarized as shown by Ki67 staining **(C**). The increase in Ki67^+^ cells (B-cells and helper T-cells) within the GC in SIV-infected macaques was concomitant with T-cell activation in the LP and T-cell zones. Whereas rare CD68^+^ macrophages were present within B-cell follicles in controls, they were consistently present in GC at 14 and 28 dpi. We observed similar changes for CD23 and Ki67 staining in terminal ileum (*data not shown*). Click here for file
