# Supplementary figures and images for: Default in plasma and intestinal IgA responses during acute infection by simian immunodeficiency virus
Source: Retrovirology. 2012 May 25;9:43. doi: 10.1186/1742-4690-9-43 (PMC3414759; doi:10.1186/1742-4690-9-43)

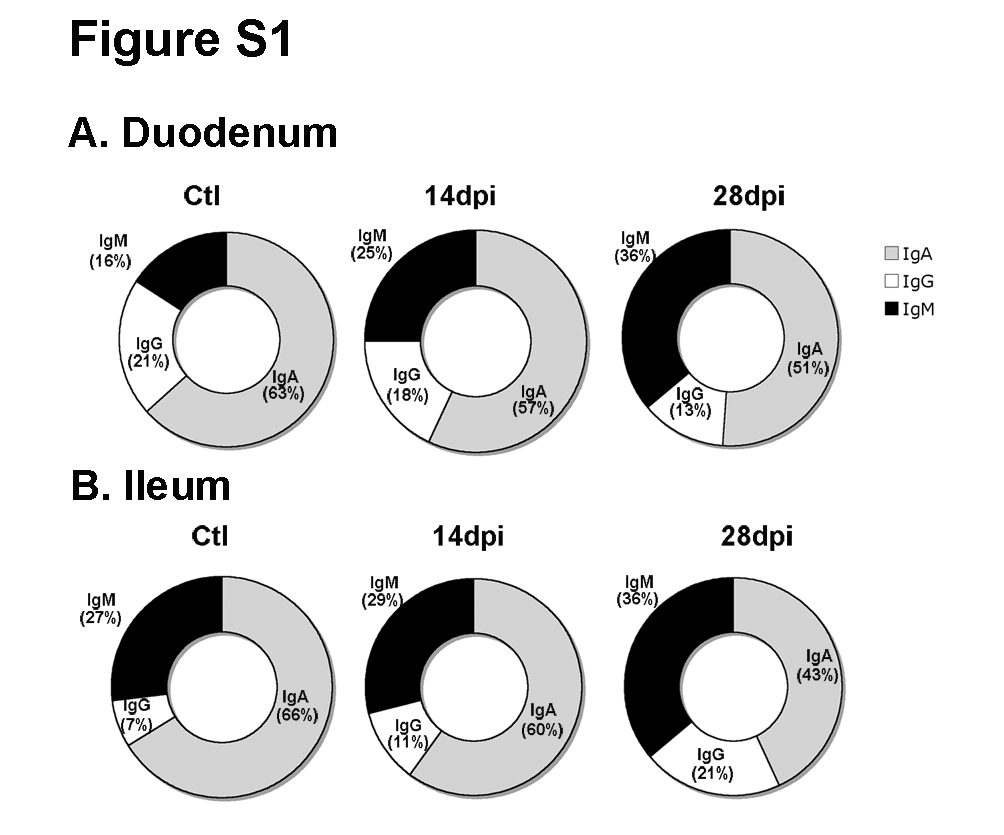

Supplement: Additional file 1 — Figure S1. Acute SIV-Infection changes the density of IgA, IgG and igM plasma cells in the intestinal LP. The relative proportions of IgA, IgG and IgM plasma cells in the LP of duodenum (A) or ileum (B) were calculated, for each group of macaques, as the ratio between the median numbers of positive cells for one isotype to the median number of total plasma cells X100. [file 1742-4690-9-43-S1.tiff]

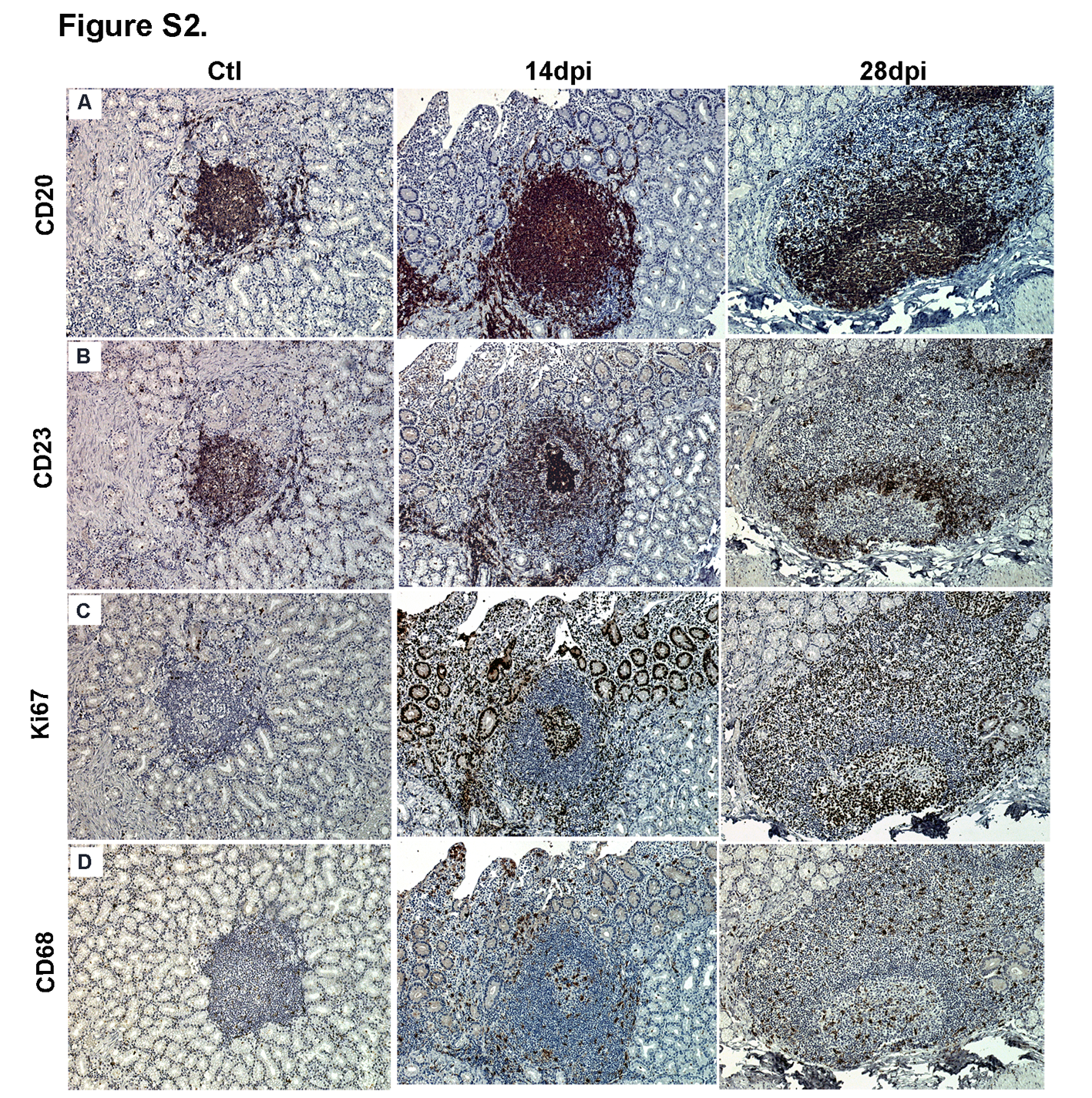

Supplement: Additional file 2 — Figure S2. Preserved polarization of GC in SIV-infected macaques. (A-D) Duodenum sections from controls (Ctl; left panel) and macaques infected for 14 dpi (middle panel) and 28 dpi (right panel) were stained with CD20 (A), CD23 (B), Ki67 (C) and CD68 (D) Ab. Stained sections from one representative macaque per group are shown. Original magnification: x100 for all panels. Because T-dependent response is strongly dependent on GC, we analyzed the GC organization after SIV infection. CD23 mAb strongly stains mature FDC network of the light zone, while Ki67 Ab stains proliferating B-cells present in the dark zone and helper T-cells in the light zone. ILF without GC in the duodenal mucosa of controls were stained by CD20 mAb (A) but not by Ki67 mAb (C). In the absence of typical GC-like structures, CD23 mAb consistently stained the network of stromal cells in these ILF (B). After SIV-infection, GC progressively developed in B-cell follicles with numerous Ki67+ cells on 14 dpi and a strong staining of a patchy FDC network. On 28 dpi, GCs were clearly hyperplasic but still correctly polarized as shown by Ki67 staining (C). The increase in Ki67+ cells (B-cells and helper T-cells) within the GC in SIV-infected macaques was concomitant with T-cell activation in the LP and T-cell zones. Whereas rare CD68+ macrophages were present within B-cell follicles in controls, they were consistently present in GC at 14 and 28 dpi. We observed similar changes for CD23 and Ki67 staining in terminal ileum (data not shown). [file 1742-4690-9-43-S2.tiff]
